# Simvastatin-Loaded Nanostructured Lipid Carriers as Topical Drug Delivery System for Wound Healing Purposes: Preparation, Characterization, and *In Vivo* Histopathological Studies

**DOI:** 10.34172/apb.2023.083

**Published:** 2023-05-20

**Authors:** Seyedeh Maryam Mousavi-Simakani, Amir Azadi, Nader Tanideh, Navid Omidifar, Parisa Ghasemiyeh, Soliman Mohammadi-Samani

**Affiliations:** ^1^Department of Pharmaceutics, School of Pharmacy, Shiraz University of Medical Sciences, Shiraz, Iran.; ^2^Stem Cells Technology Research Center, Shiraz University of Medical Sciences, Shiraz, Iran.; ^3^Department of Pathology, School of Medicine, Shiraz University of Medical Sciences, Shiraz, Iran.; ^4^Department of Clinical Pharmacy, School of Pharmacy, Shiraz University of Medical Sciences, Shiraz, Iran.; ^5^Pharmaceutical Sciences Research Center, School of Pharmacy, Shiraz University of Medical Sciences, Shiraz, Iran.

**Keywords:** Simvastatin, Nanostructured lipid carriers, Topical drug delivery systems, Histopathological studies, Wound healing

## Abstract

**Purpose:**

Simvastatin, a 3-hydroxy-3-methylglutaryl coenzyme A (HMG-CoA) reductase inhibitor, is a commonly used drug to reduce total cholesterol and low-density lipoprotein (LDL) levels. Furthermore, several mechanisms showed the wound-healing potential of statins, especially simvastatin. Simvastatin is a lipophilic drug, therefore, it has low water solubility with limited skin permeability potential. In this regard, nanostructured lipid carriers (NLCs) were recruited as novel topical drug delivery systems to enhance skin adhesion and film formation, maintain skin integrity, sustain the release of simvastatin, and prolong simvastatin skin deposition to help pressure ulcers healing and regeneration.

**Methods:**

NLCs were fabricated using the solvent diffusion evaporation technique. Drug loading, *in vitro* drug release, and morphological assessment on the optimized formulation were considered. Furthermore, *in vivo* effect of simvastatin-loaded NLCs gel on pressure ulcer healing was assessed using a rat skin model. Histopathological assessments were compared with conventional simvastatin gel and drug-free NLCs gel.

**Results:**

Simvastatin-loaded NLC with an average diameter of 100 nm was considered as the optimum formulation. According to the results entrapment efficiency of simvastatin within the NLCs was about 99.4%. Drug release studies revealed sustained drug release from NLCs in which about 87% of the drug was slowly released during 48 hours. Animal study results confirmed that simvastatin-loaded NLCs gel has better efficacy on pressure ulcers and could significantly reduce inflammation, and promote skin regeneration compared to both drug-free NLCs and conventional simvastatin gels.

**Conclusion:**

Simvastatin-loaded NLCs with an average particle size of 100 nm would be a promising novel topical drug delivery system with sustained drug release potential for pressure ulcer treatment.

## Introduction

 A wound is defined as a disorder of the lining of the skin or mucus which is caused by physical, chemical, or thermal damage. According to the duration and nature of the healing process, wounds can be classified into 2 main categories including acute and chronic wounds.^1^ Acute wounds can be induced by trauma or surgical intervention, while chronic incurable ulcers generally can be divided into four main classes including pressure ulcers, diabetic ulcers, venous ulcers, and ischemia-induced ulcers (arterial failure).^1^ The main focus of this study is on pressure ulcers which are defined as local areas of tissue necrosis caused by prolonged pressure, leading to local damage. Pressure ulcers affect 1 to 5% of inpatients and up to 26.2% of patients transferred to emergency departments from nursing homes.^2^ Since the skin layers especially the stratum corneum layer acts as a protective barrier against the environmental stresses, any injury in it must be repaired quickly and efficiently. In this regard, specific attention to wound healing processes would be crucial.^3^ Wound healing is a dynamic process consisting of several continuous and planned phases. The events of each stage must happen in a precise and regulated manner. Interruptions or prolongation in this process can lead to delay in wound healing or incurable chronic wound induction. In adults, the optimal wound healing stages include rapid homeostasis, appropriate inflammation, differentiation, proliferation, and migration of mesenchymal cells to the wound site, appropriate angiogenesis, regrowth of epithelial tissue on the wound surface, and finally, proper synthesis, cross-linking, and collagen alignment to strengthen the improved tissue.^4-8^

 Statins, also known as 3-hydroxy-3-methylglutaryl coenzyme A (HMG-CoA) reductase inhibitors are commonly used in lowering total cholesterol and low-density lipoprotein (LDL) levels.^9^ Furthermore, it has been reported that statins can prevent ischemic damage-induced angiogenesis in animals that are neuro cholesterolemic.^10^ Simvastatin, a white crystalline powder,^11^ is a lipophilic drug with a log P of 4.46.^12^ In addition to its hypocholesterolemic effect, it has been reported that simvastatin can enhance vascular endothelial growth factor synthesis and release at the wound site, which is a vital stage in the wound healing process.^13^ Furthermore, simvastatin can promote epithelialization and strengthen the epidermal barrier of normal skin, by reducing the isoprenylation of mevalonate and farnesyl pyrophosphate (FPP). Reducing FPP levels could increase keratinocyte migration and epithelialization and wound closure in a living human wound model.^10^

**Figure 1:**
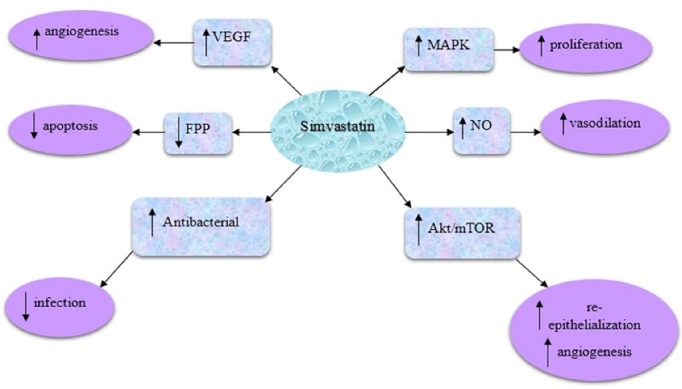
The possible mechanisms of simvastatin as a promising agent in pressure ulcer healing processes

 The possible mechanisms of simvastatin in the wound healing process are shown in Figure 1. In this regard, simvastatin can stimulate the mitogen-activated protein kinase factor which is effective in skin proliferation processes. Also, simvastatin can enhance nitric oxide levels, which is crucial in the vasodilation process. In addition, simvastatin can stimulate the vascular endothelial growth factor and therefore enhance the angiogenesis process. Furthermore, it is capable to inhibit the conversion of mevalonate to isopentenyl pyrophosphate. As a result, isopentenyl pyrophosphate conversion to geranyl pyrophosphate is inhibited, and therefore, FPP is not synthesized and the apoptosis process can be inhibited accordingly. In addition, a reduction in FPP level can be useful in the increment of keratinocyte migration, epithelialization, and wound closure process.^14^ Simvastatin can also activate the protein kinase B/mammalian target of the rapamycin (Akt/mTOR) pathway, which can result in increased angiogenesis, collagen deposition, and re-epithelialization at the wound site and therefore lead to wound healing occurrence.^15^ Last but not least, simvastatin has shown both anti-inflammatory and antibacterial properties on open wounds.^16^

 According to the consequence of the aforementioned mechanisms of simvastatin in wound healing processes, in this study, simvastatin has been considered as a promising drug to treat pressure ulcers. The topical route of drug administration would be the first choice in the management of various skin disorders. However, the main drawback of this route of administration is the presence of a stratum corneum layer. Therefore, different nanoparticulate drug delivery systems including lipid nanoparticles,^17^ vesicular nanocarriers,^18^ hydrogels,^19^ and polymeric nanoparticles^20^ were recruited to enhance skin deposition and obtain targeted drug delivery to skin organelles.^21^ Since conventional topical formulations of simvastatin showed limited efficiency in wound healing, in this regard, nanostructured lipid carriers (NLCs) were used as novel topical drug delivery systems with the advantages of enhanced skin adhesion and film formation, maintenance of skin integrity, sustained drug release, enhanced dermal bioavailability, and prolonged skin deposition to help pressure ulcers healing and regeneration.^22^ In addition, targeted drug delivery to the skin layers with an extended drug release profile can lead to the limited systemic absorption and enhanced local concentration of active pharmaceuticals.^23,24^

 NLCs are the second generation of lipid nanoparticles that are made of a mixture of solid and liquid lipids, and also surfactants. The presence of liquid lipids in NLCs can result in defection and imperfection in their structure with a less crystalline matrix which in turn can lead to enhanced drug loading potential, reduced burst drug release, and enhanced physicochemical stability in comparison to solid lipid nanoparticles as the first generation of the lipid nanoparticles that are solely fabricated of solid lipids and surfactants.^25^ NLCs are considered as potential topical drug delivery systems to enhance skin deposition, targeted delivery to skin organelles, and therefore reduce the required dose of the drug for therapeutic purposes.^26^ NLCs can encapsulate both hydrophilic and lipophilic agents, however, due to their lipophilic nature, the encapsulation efficiency and loading capacity of lipophilic drugs would be higher.^27^ Therefore, NLCs can be considered as suitable nanocarriers for simvastatin which is a lipophilic agent with a log P of 4.46. In addition, lipid nanoparticles, especially NLCs, are considered as promising topical drug delivery systems for wound healing purposes due to their biocompatibility and biodegradability characteristics, non-cytotoxic and non-irritant nature, long-term physicochemical stability, and occlusive properties that can lead to maintaining wound moisture to accelerate the wound healing process.^28^

 In this study, simvastatin-loaded NLCs with desired particle size were fabricated and characterized. A topical gel formulation of simvastatin-loaded NLCs was prepared and its wound healing properties were assessed in the animal model in comparison to the drug-free NLCs gel and conventional simvastatin gel. The main purpose of this study was to design and evaluate a novel topical drug delivery system for targeted simvastatin delivery to the pressure induced wound skin to accelerate the wound healing process, induce controlled drug release, enhance adhesion, skin deposition, and local drug concentration at the targeted area, reduce systemic absorption, and prevent further adverse drug reactions.

## Materials and Methods

###  Materials

 Cholesterol, stearic acid, lactose, sodium hydroxide, sodium chloride, potassium chloride, potassium dihydrogen phosphate, disodium hydrogen phosphate, Tween 80, propylparaben, methylparaben, and formalin were obtained from Merck, Darmstadt, Germany. Acetone, acetonitrile, and methanol were HPLC grade and purchased from Merck’s domestic supplier in Iran. Simvastatin was from Artemis, India. Brij 35 and triolein were obtained from Sigma, USA. Brij 72 was from Fluka, England. Capryol PGMC was purchased from GATTEFOSSE, France. Sodium carboxymethyl cellulose (NaCMC) was obtained from SAMCHUN, South Korea. Carbomer 934 was from BF Good Reach, USA. Ketamine 10% was obtained from BREMER PHARMA GMBH, Germany. Xylazine was from Bioveta Company, Czech Republic. Gentamicin 5% was gifted by Royan Darou, Iran, and oxytetracycline spray was from Aras Bazar Pharmaceutical Lab, Iran.

###  Statistical analysis

 During this research, statistical assessments were performed using SPSS software (version 25, 2017). The repeated measures ANOVA test was used and *P* values < 0.05 were considered as statistically significant.

###  Quantitative analysis

 All experiments were accomplished in triplicate and mean ± SD was reported. Drug analysis was performed by a validated high-performance liquid chromatography (HPLC; Agilent Technologies 1260 infinity, USA) method. The mobile phase was acetonitrile: water: methanol (70:20:10) with a flow rate of 1 ml/min. The retention time was 7 minutes. Method validation was done for simvastatin. In this method, the R square was 0.9999 which revealed acceptable linearity. The limit of quantification (LOQ) and limit of detection (LOD) were 1 μg/mL and 0.33 μg/mL respectively. Also, between and within-day precision values were less than 10% and accuracy values were between 90 and 110%.

###  Simvastatin-loaded NLCs preparation 

 Lipid nanoparticles were prepared using the solvent diffusion evaporation technique according to the previously reported technique.^29,30^ Briefly, stearic acid, Capryol PGMC, cholesterol, triolein (50:30:10:10 w/w ratio), and simvastatin were completely dissolved in acetone as an organic phase. Surfactants including Brij 35 and Brij 72 (1:1 w/w ratio, HLB_M_ = 10.91) were dissolved in distilled water and heated up to 70 °C using a water bath. Then, the organic phase was added to the aqueous phase (1:2 v/v ratio) at a predetermined mixing rate and addition time.^24^ The stirring was continued until the volume of the sample was reduced to one-third and the smell of acetone was completely removed. Then the prepared sample was transferred to an ice bath (below 5 °C) to solidify lipid nanoparticles. Particle size was freshly measured using a particle size analyzer (PSA, SALD-2101, SHIMADZU, Japan), and also zeta potential was assessed by Zeta-check (Microtrac, ZC007, Germany).

###  Simvastatin-loaded NLCs characterization 

####  Particle size and size distribution analysis 

 Particle sizes of nanoparticles were measured freshly and 1 month later using a PSA. In order to prove the PSA data, dynamic light scattering (DLS, SZ-100, Horiba, Japan) and transmission electron microscopy (TEM, LEO 906 E, Zeiss, Germany) were also considered. The span index was calculated as a measure of polydispersity according to Eq. 1.


(Eq. 1) $$Span\;index=\frac{D90-D10}{D50}\times 100$$

Where D90, D50, and D10 are 90%, 50%, and 10% undersized diameters, respectively.

####  Physical stability assessment

 The physicalstability of the NLCs was evaluated in terms of particle size change during of the time and drug expulsion assessment for 2 months (samples were refrigerated at 2-8 °C). At first, simvastatin was added as 20%, 15%, 10%, and 5% of total lipid, respectively. However, due to drug expulsion and particle size increment during 7 days after storage condition, the percentage of simvastatin was reduced to 2% of total lipid content. In this regard, the optimum formulation (containing 50% stearic acid, 30% Capryol PGMC, 10% triolein, 10% cholesterol, and 2% simvastatin) was selected as optimum formulation for further evaluations, and drug expulsion was assessed for at least 2 months.

####  Drug loading assessment

 Simvastatin was added to the organic phase and simvastatin-loaded NLCs with an average diameter of 100 nm were prepared with the solvent diffusion evaporation technique as mentioned previously. Five milliliters of each prepared sample were poured into the upper chamber of centrifugal filter tubes (MWCO 10 kDa, Amicon Ultra-4, Millipore Co, MA, USA) and centrifuged at 4000 rpm for 15 minutes. Filtrates were analyzed by a validated HPLC method (Agilent Technologies 1260 Infinity, USA) to measure unloaded drugs. All experiments were repeated three times. The percentages of entrapment efficiency (%EE) and drug loading capacity (%LC) were calculated according to Eq. 2 and Eq. 3, respectively.


(Eq. 2) $$\%EE=\frac{Loaded\;Drug}{Total\;Drug}\times 100$$

The total drug is the amount of drug (mg) which was initially added to the organic phase and the loaded drug (mg) was calculated through the subtraction of the unloaded drug from the total drug.


(Eq. 3) $$\%LC=\frac{Loaded\;Drug}{Total\;Lipid+Loaded\;Drug}\times 100$$

Loaded drug (mg) is total drug minus unloaded drug as mentioned and total lipid (mg) is the mixture of lipids used in the organic phase.

####  Transmission electron microscopy (TEM)

 The morphology and size of simvastatin-loaded NLCs were investigated by TEM (LEO 906 E, Zeiss, Germany). To do so, at first, simvastatin-NLC was diluted with water (1:5 ratios), then, one drop of the sample was fixed in copper grids and stained with uranyl acetate to visualize lipid nanoparticles.

####  In vitro drug release assessment using the dialysis membrane

 Drug release assessment was performed using dialysis membrane (Dialysis tubing cellulose membrane, D9652-100FT, Sigma-Aldrich, cut off 14 000 Da). Drug release from NLCs with an average diameter of 100 nm was evaluated in triplicate and results were compared with the permeation of free drug through the dialysis membrane. The release medium was 100 mL of a mixture of 98% phosphate buffer saline (PBS, 10 mM, pH 7.4) and 2% Tween 80 to maintain sink condition. The temperature of the release medium was fixed at 37 °C and the medium was mixed continuously at 150 rpm. In this regard, 16 mL of the freshly prepared NLCs was condensed to 3 mL (containing 2.12 mg simvastatin) and poured into the dialysis membrane, and then floated in the release medium. Sampling (500 μL) was performed at 0, 0.5, 1, 2, 4, 8, and 24 hours and the release medium volume was kept constant by immediately replacing the same volume of the fresh release medium (37 °C). In the end, the prepared samples were analyzed by a validated HPLC method.

####  In vitro drug release assessment by centrifugation method 

 In this regard, 750 μL of simvastatin-loaded NLCs was mixed with 4.25 mL of release medium and the mixture was placed in a shaker-incubator at 37 °C with a mixing rate of 100 rpm. 18 tubes containing 5 mL of the mixture of release medium and simvastatin-loaded NLCs were stored in the aforementioned conditions for 48 hours. After 1, 2, 4, 8, 24, and 48 hours, three of these mentioned tubes were taken and poured into Amicon filter tubes and centrifuged at 4000 rpm for 5 min. Then the filtrate was analyzed through a validated HPLC method and the amount of the released drug was calculated. After that, drug release patterns and release kinetics were assessed accordingly.

####  Differential scanning calorimetry assessment

 Differential scanning calorimetry (DSC) analysis (DSC-TGA 1, Mettler Toledo, Swiss) was performed to assess the thermal behavior and polymorphic transition of lipids in prepared NLCs and also to investigate the possible interaction of various lipids, surfactants, and simvastatin. In this regard, 90 µL of the simvastatin-loaded NLCs (containing 5 mg of lipid mixture) was used. The scan rate was fixed at 5°C/min and the samples were heated up from 25 to 90 °C. The mentioned process was performed for simvastatin-loaded NLCs, drug-free NLCs, a physical mixture of lipids (stearic acid, cholesterol, triolein, and Capryol PGMC with a 50:10:10:30 w/w ratio), and a physical mixture of lipids plus simvastatin. The results of the thermal analysis were compared with that of the simvastatin-loaded NLCs thermogram.

###  Simvastatin-loaded NLCs gel preparation

 Simvastatin-loaded NLCs gel (0.1% w/w, pH of 6.8) with average diameters of 100 nm was prepared to assess the *in vivo* effects of simvastatin-loaded NLCs topical gel on wound healing processes. For this purpose, Simvastatin-loaded NLCs with an average diameter of 100 nm were prepared and mixed with lactose as a cryoprotectant (5-fold of total lipid and 1:1 v/v ratio). After that, the mixture was lyophilized through a freeze dryer (Alpha 2-4 LDplus, Christ, Germany) and the obtained solid powders were levigated and completely dispersed in a previously prepared gel mixture containing 0.5% w/v Carbomer 934 and 2% w/v NaCMC.

###  Animal study 

 Twenty adult male Wistar rats weighing 180-240 g were used in this study. The animals were housed in groups in a temperature-controlled room with a 12:12 hour light-dark cycle with free access to rodent diet and water.

####  In vivo histopathological studies on pressure ulcers

 In order to evaluate the *in vivo* effect of simvastatin-loaded NLCs on pressure ulcer treatment, the skin behind the neck and back of the rats was used. In this regard, rats were anesthetized using a mixture of ketamine (100 mg/kg) and xylazine (10 mg/kg). In this regard, for those with an average weight of 200 g, 0.4 mL of ketamine vial (50 mg/mL) and 0.02 ml of xylazine vial (100 mg/mL) were utilized. Therefore, a volume ratio of 20:1 was used for the ketamine: xylazine mixture to induce anesthesia in rats. Then, the hairs of the aforementioned skin areas were shaved with an electric razor. The proposed skin area was disinfected using betadine. Then, the skin was cut about 2 cm using a surgical blade and surgical scissors. Using forceps, the gold-coated steel plate was placed under the skin of the rats’ necks. After that, the animals’ skin was stitched. The next step was the induction of pressure sores using 700-g weights and putting them on the skin of rats’ necks. In this regard, the animals were anesthetized through the previously mentioned protocol and then were slept side by side and the skin of the neck were located under the gold-coated steel plate and an external magnet was placed there. Finally, the aforementioned weights were placed vertically on the rats’ skins to form pressure ulcers. Therefore, magnets and weights were placed on the skin surface of the animals and the gold-coated steel plate was placed under their skin. This method was applied for 5 cycles in duration of 2 hours daily. All rats were unconscious during the procedure and they were covered with sterile gauze soaked in normal saline serum to prevent suffering from dry eyes. The magnitude of damage to the skins of the animals increased over the days and finally, on the last day, the skin necrosis was completely clear and pressure sores were generated.

 After the induction of pressure sores, the rats were anesthetized again and the plate was removed from the animals and the skin was stitched. Then rats were divided into 4 different groups; the first group was considered as the negative control group and received no treatment, the second group received the conventional simvastatin gel (0.1% w/w), the third group received the drug-free NLCs gel, and the fourth group received the simvastatin-loaded NLCs gel (0.1% w/w). The duration of pressure ulcer treatment was followed for 2 weeks and the prepared gels were applied twice daily at the morning and evening on the wounded area. After two weeks, the rats were sacrificed and the skin of the damaged area, which was treated with various topical gel formulations was cut and stored in a formalin solution of 10% for one week in open-lid dishes. In the end, the skins samples were evaluated.

 After appropriate sample preparation from skin tissues, a paraffin block was prepared from the samples. Then, the slides were prepared with a diameter of 4 to 6 µm thickness and stained with the hematoxylin and eosin (H&E) technique.^31^ Then, the prepared slides were evaluated by a pathologist through an optical microscope.

## Results and Discussion

###  Particle size and size distribution

**Figure 2:**
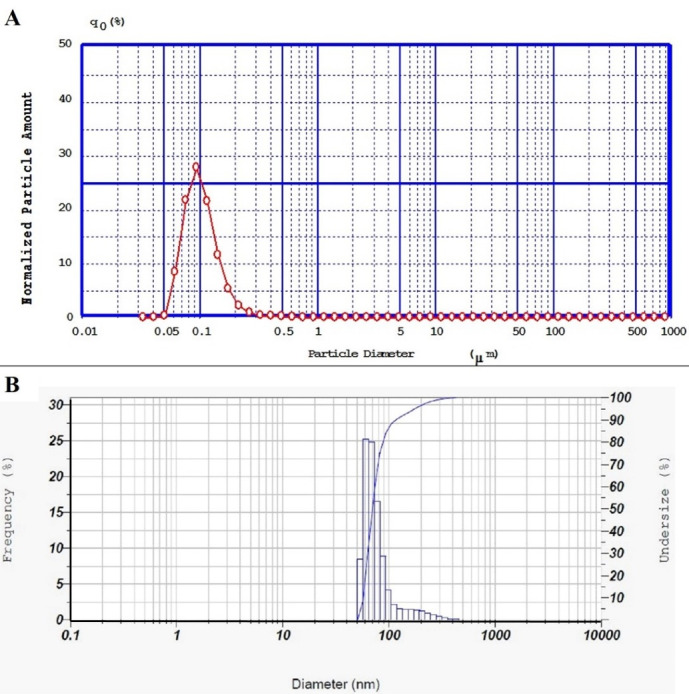
(A) PSA diagram of simvastatin-loaded NLCs with an average diameter of 100 nm and span index of 0.89, (B) DLS diagram of simvastatin-loaded NLCs with an average diameter of 83 nm and polydispersity index (PDI) of 0.35

 Particle size and size distribution analysis confirmed that nanoparticles are homogenous in size with an acceptable low span index (0.89) and polydispersity index (0.35). A typical graph of DLS and PSA of simvastatin-loaded NLCs was shown in Figure 2. Furthermore, the zeta potential for these nanoparticles was -13.8 ± 0.889 mV, this negative charge was in agreement with previous research on some lipid nanoparticles.^32^ The negative zeta potential of the prepared NLCs can provide electrostatic repulsion among lipid nanoparticles, so the storage stability of the fabricated nanoparticles could be enhanced.

###  Stability studies

**Figure 3:**
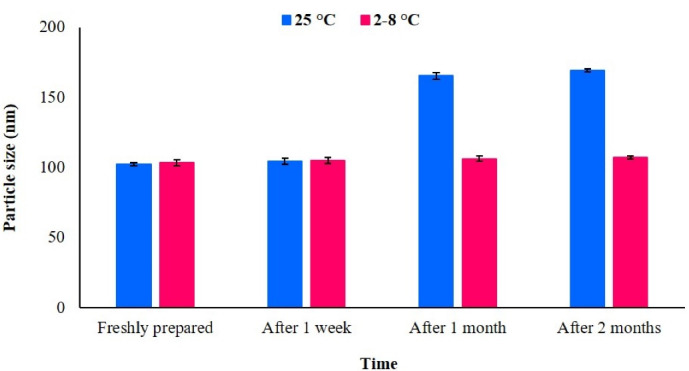
Particle size stability test at room temperature and in the refrigerator (2-8 °C); just after formulation preparation up to 2 months later

 As shown in Figure 3, simvastatin-loaded NLCs particle size was stable for up to 2 months in the refrigerator (2-8 ˚C). However, the particle size was enhanced at room temperature after 2 months. Drug expulsion assessments revealed that in optimum formulation (containing 50% stearic acid, 30% Capryol PGMC, 10% triolein, 10% cholesterol, and 2% simvastatin), no expulsion occurred during the 2-month storage.

###  Drug loading 

 The mean percentage of drug entrapment efficiency and loading capacity of the targeted particle size (100 nm), which was assessed through the centrifugation ultrafiltration technique, were 99.43 ± 0.163% and 1.95 ± 0.003%, respectively.

 Simvastatin is a highly lipophilic drug (log P = 4.46) and is practically insoluble in water; so, it would be anticipated that exhibits high entrapment efficiency (99.43 ± 0.16%) in lipid nanoparticles. Also, according to the previous reports NLCs in comparison to solid lipid nanoparticles have a higher drug loading capacity and entrapment efficiency due to the presence of liquid lipids and enhanced drug solubility in the lipid matrix.^24,33^ based on the results in this nanoparticulate drug delivery system, no drug expulsion has occurred during 2 months of the storage stability period when the levels of the simvastatin reduced to 2 % of lipids level. Although the percentage of the loading capacity of simvastatin within NLCs was relatively low (1.95 ± 0.003%), however, due to the high potency of simvastatin and also the sustained release pattern of the simvastatin at the site of the ulcer it can accompany sufficient clinical outcomes.^14,16^

###  Transmission electron microscopy 

**Figure 4:**
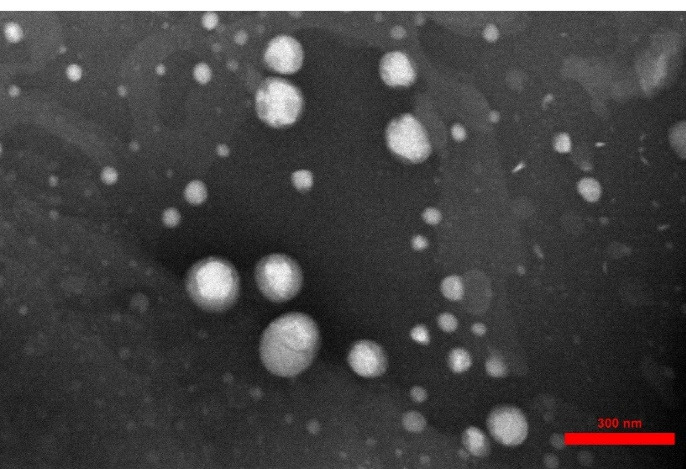
TEM of simvastatin-loaded NLCs with an average diameter of 100 nm

 Morphological assessment of simvastatin-loaded NLCs revealed that they were spherical and relatively uniform in shape as shown in Figure 4. TEM results were in agreement with the results of PSA and DLS data in terms of average particle size.

###  In vitro drug release 

**Figure 5:**
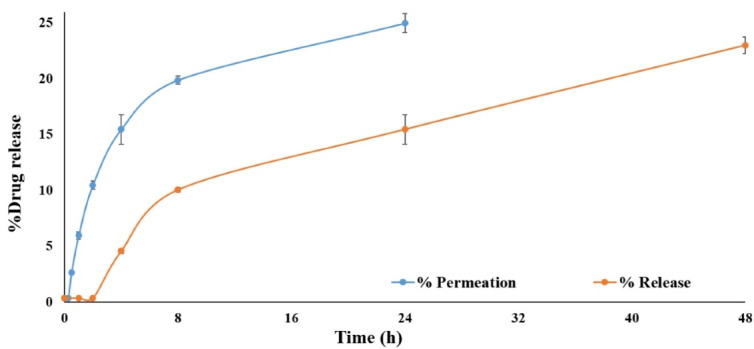
The drug release pattern of simvastatin-loaded NLCs in comparison to the free drug permeation through the dialysis membrane

**Figure 6:**
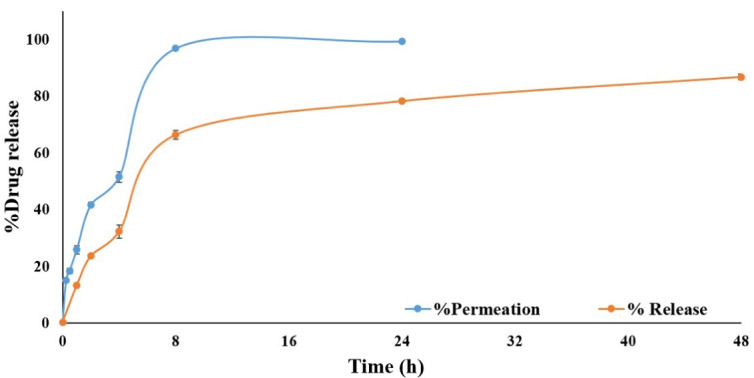
The drug release pattern of simvastatin-loaded NLCs in comparison to the free drug through the centrifuge method

 Drug release from simvastatin-loaded NLCs with an average diameter of 100 nm was assessed through the dialysis membrane for 48 h and the obtained results were compared with the permeation of the free drug through the dialysis membrane as demonstrated in Figure 5. The results revealed that simvastatin release from NLCs was significantly slower (*P* value < 0.05) in comparison to the permeation of free drugs through the membrane. Since only up to 25% of the free drug permeated through the dialysis membrane during 24 hours and only 20% of simvastatin was released from NLCs and permeated through the membrane during 48 hours, therefore, centrifugation method was also used for drug release assessment. In this regard, the effect of the drug release assessment technique on the permeation rate of simvastatin was assessed. According to the release experiment results, the application of different techniques could significantly affect the final results. As it is clear in Figure 6, the permeation rate of the free drug was profoundly increased in the centrifugation technique and 100% of the drug was permeated within 8 hours and reached a plateau during 24 hours. In addition, up to 80% of the simvastatin was slowly released from NLCs and permeated during 48 hours which was significantly (*P* value < 0.05) higher than that of the dialysis membrane technique. The simvastatin release rate from the NLCs was significantly slower than the permeation of the free drug which confirmed the sustained release pattern of simvastatin from the NLCs with an average diameter of 100 nm. Since the purpose of the design and development of this novel drug delivery system was to induce localized effects at the wound site with minimal systemic absorption, this sustained drug release pattern can accompany prolonged drug deposition at the target site with minimum adverse reactions.

 Results of a previous study on simvastatin-loaded NLCs for vitiligo management reported an 80% drug release during 8 hours, therefore the current study was accompanied by more sustained drug release during 48 hours which can lead to the enhanced interval of dosage form application. This can be attributed to the differences in lipid matrix nature and also type and concentration of surfactants in NLCs design and fabrication could profoundly affect the release pattern.^34^ Another study on tissue scaffolds of simvastatin-loaded NLCs showed an initial burst release followed by an extended-release during 24 hours.^35^

 Drug release kinetics was assessed using the centrifugation technique. According to the results of drug release kinetics (Table 1), simvastatin release from NLCs was best fitted to the Higuchi model with the highest R-squared value (0.922). The Higuchi model is a suitable kinetic model for matrix-based drug delivery systems as reported in previous studies.^36^ Although, the Korsmeyer-Peppas model showed an R-squared value of 0.938, however, this model is most commonly used to determine the mechanism of drug release from nanoparticles. According to the Korsmeyer-Peppas equation, the n value (release exponent) of 0.576, which is between 0.5 and 1, revealed that the mechanism of drug release from NLCs followed a non-Fickian or anomalous transport in which simultaneous erosion and diffusion has occurred simultaneously during simvastatin release from nanoparticles although it seems that the weight of the diffusion in more prominent.^37^

**Table 1:** Simvastatin release kinetics from NLCs

**Kinetic model**	**Zero-order**	**First-order**	**Higuchi**	**Korsmeyer-Peppas**	**Hixon-Crowell**
R-squared	0.765	0.871	0.922	0.938 (n = 0.576)	0.838

###  DSC analysis 

**Figure 7:**
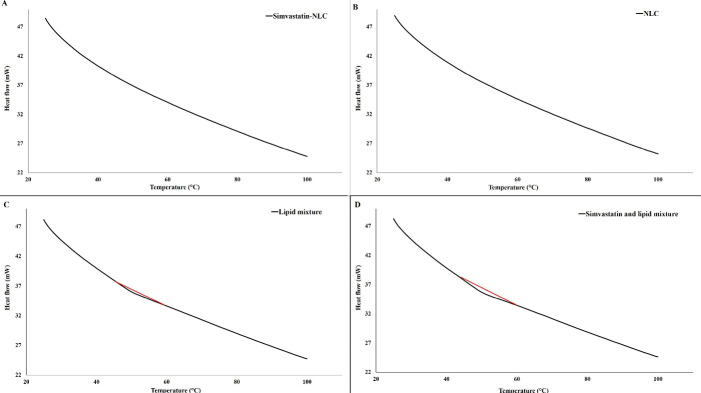
(A) DSC thermogram of simvastatin-loaded NLCs, (B) DSC thermogram of drug-free NLCs, (C) DSC thermogram of physical mixture of lipids, and (D) DSC thermogram of physical mixture of lipids and simvastatin

 The DSC thermograms of simvastatin-loaded NLCs, drug-free NLCs, a physical mixture of lipids, and a physical mixture of lipids plus simvastatin were shown in Figure 7. Based on Figure 7, no clear endothermic or exothermic changes were observed in the simvastatin-loaded NLCs and drug-free NLCs thermograms (Figure 7A and Figure 7B), indicating that the prepared NLCs had an amorphous nature and no crystallization was in lipid components take a place. Also, the DSC results of our previous study on NLCs with the same lipid matrix and surfactants revealed that there was no interaction between NLCs ingredients.^23^ Moreover, the current study revealed that there was no obvious interaction between drug and NLC ingredients and also lipid mixture. In addition, due to the presence of a small endothermic peak in the thermogram of the physical mixture of lipids, it can be concluded that NLCs had different thermal behavior than the physical mixture of lipids, which can be attributed to the changes in the arrangement of the lipid chains during the process of nanoparticle fabrication and amorphous matrix formation.^23^

###  In vivo effect of simvastatin-loaded NLCs on pressure ulcers 

**Figure 8:**
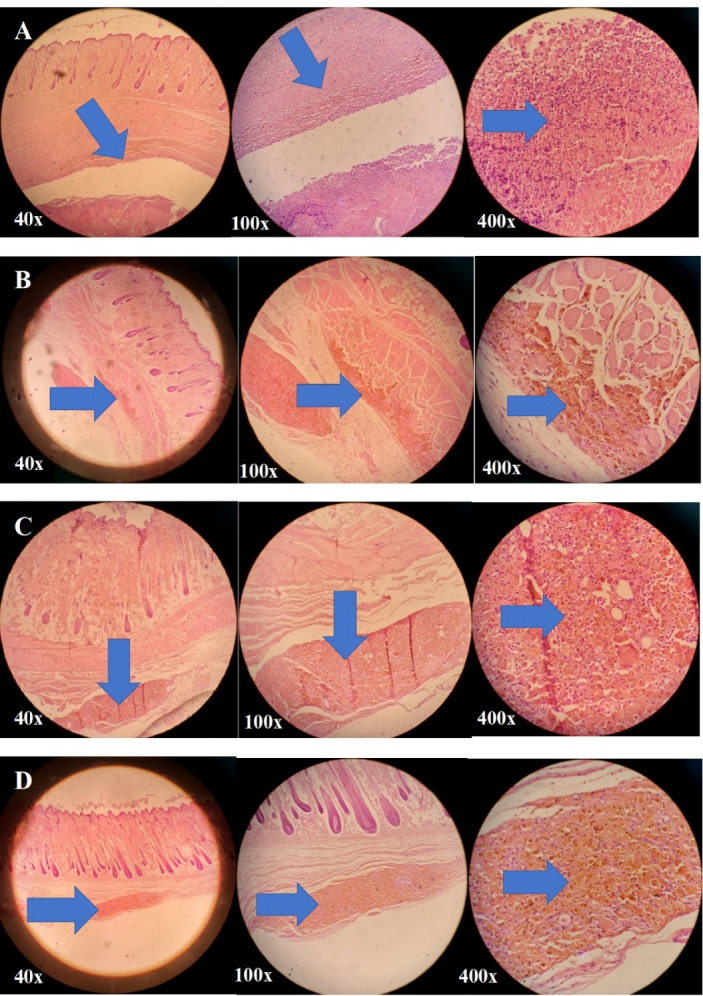
Microscopic images of (A) the damaged rat skin of the negative control group, the group without any treatment after 2 weeks; the injury rate of + + + , (B) rat skin of the second group treated with conventional simvastatin gel 0.1% for 2 weeks; the injury rate of + + , (C) rat skin of the third group treated with drug-free NLCs gel for 2 weeks; the injury rate of + + , and (D) rat skin of the fourth group treated with simvastatin-loaded NLCs gel 0.1% for 2 weeks; the injury rate of + ; the affected areas in all these images are marked with arrows

 In order to investigate the beneficial effect of simvastatin-loaded NLCs on pressure ulcer treatment, rats were divided into 4 different groups. In the first group (negative control group), animal did not receive any treatment. At the end of the 2 weeks period, this group had a slow wound repair process, and also inflammation, hemosiderin granule formation, and infiltration were prominent with an injury magnitude of + + + . In the second group, rats were treated with topical conventional simvastatin gel 0.1% for 2 weeks. According to Figure 8, after two weeks of wound formation, inflammation, hemosiderin granule reabsorption, infiltration, and granulation formation of tissues were observed moderately. The epidermis and dermis layers were almost completely healthy; however, the hypodermis layer was somewhat damaged (injury magnitude of + + ). In the third group, rats were treated with drug-free NLC gel for 2 weeks. According to Figure 8, after two weeks of wound formation, inflammation, hemosiderin, infiltration, and granulation formation of tissues were moderately noticeable. The epidermis and dermis were almost completely healthy, but the hypodermis was somewhat damaged (injury magnitude of + + ). In the fourth group, rats were treated with simvastatin-loaded NLC gel 0.1% for 2 weeks. According to Figure 8, after two weeks of treatment, infiltration, and hemosiderin granules were significantly lower in comparison to the other third groups. The epidermis and dermis layers were completely intact, however, the hypodermis layer was partially involved (injury magnitude of + ). The qualitative pathological evaluation was performed by an expert pathologist.

 Therefore, according to Figure 8, the second and third groups of the rats that were treated with conventional simvastatin gel 0.1% and drug-free NLCs gel, respectively, showed almost the same levels of improvement in pressure ulcers and the therapeutic effect of these two formulations were almost identical. So, both drug-free NLCs and conventional simvastatin gels were effective in the wound healing process probable due to the occlusive and dressing properties of these formulations although their effectiveness was less than the fourth group. The fourth group, which was treated with simvastatin-loaded NLCs gel 0.1%, had the best recovery rate with the lowest injury magnitude of + after 2 weeks of treatment and showed the best results for wound healing purposes.

 Previous studies on wound healing properties of simvastatin showed that there was no significant difference between the group of rats treated with HPMC/chitosan gel containing simvastatin and those treated with conventional simvastatin gel.^38^ Results of another study on formulations containing various amounts of simvastatin revealed that increasing the percentage of the drug in formulations was accompanied by a higher recovery rate of induced wounds. However, it was suggested that more rats should be recruited to obtain more accurate and reliable conclusions.^39^ Based on the results of the present study, it seems that simvastatin-loaded NLCs gel 0.1% with an average diameter of 100 nm would be a promising formulation for wound healing purposes due to the sustained drug release potential, and prolonged drug deposition at the site of action, and optimum beneficial histopathological effects on pressure ulcers healing.

## Conclusion

 NLCs are considered as suitable topical drug delivery systems with numerous advantages. The most important advantages of these drug delivery systems are their biocompatibility, biodegradability, controlled drug release potential, long-term physicochemical stability, and convenience of industrial-scale production. Furthermore, lipid nanoparticles are suitable nanocarriers for both lipophilic and hydrophilic drugs. Simvastatin is a lipophilic drug with limited skin penetration potential due to its low water solubility and high log P. In this regard, NLCs were recruited as suitable nanocarriers for targeted delivery to the site of pressure ulcers, enhancing dermal bioavailability and skin deposition, and controlling the drug release rate at the site of action. Therefore, simvastatin-loaded NLCs with an average particle size of 100 nm were investigated in this study. The results revealed that this novel topical drug delivery system has high entrapment efficiency, sustained drug release characteristics, and prolonged deposition at the wound site, and also showed promising histopathological results confirming superior healing properties in pressure ulcer treatment. However, the recruitment of a positive control group in the animal wound-healing model in future studies can be helpful to compare its wound-healing potential with that of the current study formulation. Moreover, further clinical trial studies are required to assess the efficacy of the fabricated topical simvastatin-loaded NLCs gel formulation in the wound healing process in human.

## Acknowledgments

 This study was a part of Pharm.D. thesis of Dr. Seyedeh Maryam Mousavi-Simakani. The authors would like to appreciate the financial support of the Vice Chancellor for Research of Shiraz University of Medical Sciences [Grant No.24148].

## Competing Interests

 Authors declare no conflict of interest.

## Ethical Approval

 Animals were treated ethically according to the protocol of the Ethics Committee of Shiraz University of Medical Sciences (Ethics code No. IR.SUMS.REC.1400.553).
